# Management of Malnutrition in Older Patients—Current Approaches, Evidence and Open Questions

**DOI:** 10.3390/jcm8070974

**Published:** 2019-07-04

**Authors:** Dorothee Volkert, Anne Marie Beck, Tommy Cederholm, Emanuele Cereda, Alfonso Cruz-Jentoft, Sabine Goisser, Lisette de Groot, Franz Großhauser, Eva Kiesswetter, Kristina Norman, Maryam Pourhassan, Ilse Reinders, Helen C. Roberts, Yves Rolland, Stéphane M. Schneider, Cornel C. Sieber, Ulrich Thiem, Marjolein Visser, Hanneke A.H. Wijnhoven, Rainer Wirth

**Affiliations:** 1 Institute for Biomedicine of Aging, Friedrich-Alexander-Universität Erlangen-Nürnberg, 90408 Nuremberg, Germany; 2 Department Nutrition and Health, University College Copenhagen, 2200 Copenhagen, Denmark; 3 Herlev and Gentofte University Hospital, 2703 Herlev, Denmark; 4 Clinical Nutrition and Metabolism, Department of Public Health and Caring Sciences, Uppsala University, 701 05 Uppsala, Sweden; 5 Theme Ageing, Karolinska University Hospital, 171 76 Stockholm, Sweden; 6 Clinical Nutrition and Dietetics Unit, Fondazione IRCCS Policlinico San Matteo, 27100 Pavia, Italy; 7 Servicio de Geriatría, Hospital Universitario Ramón y Cajal (IRYCIS), 28034 Madrid, Spain; 8 Heidelberg University Centre for Geriatric Medicine and Network Aging Research (NAR), University of Heidelberg, 69126 Heidelberg, Germany; 9 Division of Human Nutrition, Wageningen University, 6708 WE Wageningen, The Netherlands; 10 German Institute for Human Nutrition Potsdam-Rehbrücke, Department of Nutrition and Gerontology, 14558 Nuthetal, Germany; 11 Research Group on Geriatrics, Charité Universitätsmedizin Berlin, corporate member of Freie Universität Berlin, Humboldt-Universität zu Berlin, and Berlin Institute of Health, 10117 Berlin, Germany; 12 Institute of Nutritional Science, University of Potsdam, 14558 Nuthetal, Germany; 13 Department for Geriatric Medicine, Marien Hospital Herne—University Hospital, Ruhr-Universität Bochum, 44625 Herne, Germany; 14 Department of Health Sciences, Faculty of Science, and Amsterdam Public Health Research Institute, Vrije Universiteit Amsterdam, 1081 HV Amsterdam, The Netherlands; 15 Southampton NIHR Biomedical Research Centre, University of Southampton, Southampton General Hospital, Southampton SO16 6YD, UK; 16 Gérontopôle, Centre Hospitalo-Universitaire de Toulouse, 31059 Toulouse, France; 17 Nutritional Support Unit, Centre Hospitalier Universitaire de Nice, Université Côte d’Azur, 06200 Nice, France; 18 Department of Medicine, Kantonsspital Winterthur, 8401 Winterthur, Switzerland; 19 Centre of Geriatrics and Gerontology, Albertinen-Haus, Hamburg, and Chair of Geriatrics and Gerontology, University Medical Centre Eppendorf, 20246 Hamburg, Germany

**Keywords:** Geriatric patients, older persons, malnutrition, therapy, interventions

## Abstract

Malnutrition is widespread in older people and represents a major geriatric syndrome with multifactorial etiology and severe consequences for health outcomes and quality of life. The aim of the present paper is to describe current approaches and evidence regarding malnutrition treatment and to highlight relevant knowledge gaps that need to be addressed. Recently published guidelines of the European Society for Clinical Nutrition and Metabolism (ESPEN) provide a summary of the available evidence and highlight the wide range of different measures that can be taken—from the identification and elimination of potential causes to enteral and parenteral nutrition—depending on the patient’s abilities and needs. However, more than half of the recommendations therein are based on expert consensus because of a lack of evidence, and only three are concern patient-centred outcomes. Future research should further clarify the etiology of malnutrition and identify the most relevant causes in order to prevent malnutrition. Based on limited and partly conflicting evidence and the limitations of existing studies, it remains unclear which interventions are most effective in which patient groups, and if specific situations, diseases or etiologies of malnutrition require specific approaches. Patient-relevant outcomes such as functionality and quality of life need more attention, and research methodology should be harmonised to allow for the comparability of studies.

## 1. Malnutrition in Geriatric Patients

Although geriatric patients are usually at an advanced age, a geriatric patient is not defined by a specific age, but rather by a high degree of frailty and the presence of chronic disease. Functional reserves are reduced and vulnerability to stress increased as a consequence of a cumulative decline in many physiological systems during ageing. Limitations are, however, not restricted to physical functions, but may also affect mental and/or social integrity, leading to the need of comprehensive treatment to maintain or restore independence in everyday life as far as possible.

Among other typical geriatric syndromes, such as dementia, delirium, falls or incontinence, which are characterised by high prevalence, multifactorial origin and poor outcomes [1], malnutrition (i.e., protein-energy-malnutrition or undernutrition) merits attention.

### 1.1. Prevalence

Malnutrition is reported in up to 50% of older adults, although prevalence estimates vary substantially depending on the population considered, the healthcare setting, and the tool used for its assessment [2,3,4]. A recent systematic review and meta-analysis of studies using the Mini Nutritional Assessment^®^— the most widespread malnutrition screening tool for older people—has summarised the following estimates according to the setting of care: community, 3%; outpatients, 6%; home-care services, 9%; nursing homes, 17.5%; hospital, 22%; long-term care, 29%; rehabilitation/sub-acute care, 29%. In addition, a high proportion of older adults are at risk of malnutrition, with estimates ranging between 27% (community/outpatients) and 50% (all other healthcare settings) [2]. These figures are in accordance with other studies and reviews, describing an increasing prevalence of malnutrition with decreasing health and functional status and increasing dependency and disability [5,6]. Prevalence rates may however vary widely between study samples even when using the same definition in the same health-care setting [2,7].

Since low muscle mass is now included as one important phenotypic criterion in the new global definition of malnutrition [8,9], and older adults are characterised by a decline of muscle mass not only due to ageing and poor physical activity but also to a poor adaptation to nutritional deficits [10], it is reasonable to argue that prevalence estimates may be even higher than previously reported.

### 1.2. Etiology

Due to a variety of factors, older people and specifically in-patients are at increased risk of malnutrition. A decrease in food intake is common [11] and often associated with a disease, acute or chronic, which is increasing energy needs. The combination of a decrease in dietary intake and increased needs during an illness places the older person in a group of particular risk.

The decrease in food intake is often associated with a loss of the sensory abilities of taste and smell, that results in anorexia and is termed “anorexia of ageing” [12,13], but may also be caused by a poor oral health, difficulties in chewing and swallowing, side effects of pharmacological treatment, cognitive limitations, social isolation, loneliness or depression. Many acute conditions (e.g., infections, surgery) often occur on a background of chronic co-morbidities (e.g., heart failure, respiratory disease, cancer, renal failure) and increase energy needs and precipitate malnutrition in already vulnerable older people.

Besides these individual aspects, external factors such as the quality of meals, meal ambience, and the quality of (medical and nutritional) care may affect dietary intake and contribute to malnutrition, in particular in hospitals and care homes.

Many other factors may be involved [14,15,16], which may be of different relevance in different health-care settings, and may also differ from person to person. Thus, without doubt, the development of malnutrition in older persons is multifactorial and complex, and presently, only partly understood.

### 1.3. Consequences

It is well established that malnutrition is associated with increased morbidity and mortality both in acute and chronic disease and has serious implications for recovery from disease, trauma and surgery [17]. Loss of body protein resulting from insufficient protein intake or increased requirements in disease is one hallmark of malnutrition, followed by an impaired immune status and loss of muscle mass, which contribute in large part to the increased morbidity observed in malnutrition. In old and sick individuals in particular, decreased muscle mass and strength, referred to as sarcopenia, in turn lead to impaired physical status, loss of independence and increased risk of falls and subsequent fractures which have a debilitating impact on quality of life [18]. Recovery from disease is delayed in malnutrition with longer convalescence periods. Not surprisingly, malnourished patients therefore have significantly longer hospital stays with more infectious and non-infectious complications, an increased rate of unplanned readmissions to hospital and higher health resource utilisation in the outpatient setting. These consequences not only increase the burden for the individuals concerned, but ultimately also increase the economic burden for the health care system [19]. A prospective study in adults aged over 70 recently showed that adjusted healthcare costs were 714 € per year greater in patients with malnutrition or malnutrition risk compared to well-nourished patients, mainly due to hospital admission costs [20].

In summary, malnutrition is widespread in older people and represents a major geriatric syndrome with multifactorial etiology and severe consequences. In the following section, we aim to describe current approaches and evidence regarding malnutrition treatment, and to highlight relevant knowledge gaps that need to be addressed.

## 2. Management of Malnutrition

### 2.1. General Aims and Options

Nutritional interventions in the older adult may have several complementary aims [21]:

- Maintenance or improvement of nutritional status, which may replenish the protein and energy storage that is necessary to accommodate the needs induced by a metabolic stress;
- Maintenance or improvement of function and capacity for rehabilitation; this is mostly related to the muscle compartment including muscle mass. Activities of daily living but also community living may therefore be secondary aims;
- Maintenance or improvement of health-related quality of life, probably more important, compared to the reduction in mortality, than in younger adults; restoring food intake may play a direct role as being an important mediator of pleasure and well-being;
- Reduction of morbidity, including an improved outcome of underlying chronic diseases;
- Reduction of mortality as a consequence of morbidity reduction but also by increasing treatment tolerability of the underlying chronic disease (e.g., cancer);
- Reduction of malnutrition-associated costs (reduction of the hospital length of stay, the need for subacute care stays, reduction of nursing home admissions, the number of medical examinations and prescriptions).

Thus, the aims and approaches of malnutrition treatment in older patients do not generally differ from those in younger patients, but maintenance of function and quality of life gain in importance compared to reduction of mortality.

Nutritional interventions for older people cover a broad range of different measures, which all may contribute to support adequate intake and go far beyond just providing adequate amounts of energy and nutrients. As with any geriatric syndrome, the identification and management of multiple causes constitutes the basis of appropriate nutritional care. Furthermore, adequate intake may be supported by various strategies—first of all but not only by help with eating if required. Regarding direct nutritional measures, oral strategies are always the first choice. These include various modifications of usual foods as well as offering oral nutritional supplements. Moreover, enteral and parenteral nutrition are important options also for older patients, although less often indicated.

### 2.2. Current Recommendations and Evidence: ESPEN Guidelines 2019

Current knowledge about the effectiveness of nutritional interventions is summarised in the updated ’ESPEN guidelines on clinical nutrition and hydration in geriatrics’, which were developed in a multidisciplinary group of 13 experienced experts from 9 European countries [21]. In contrast to the previous guidelines from 2006 [22] and 2009 [23], they focus on systematic reviews, where available, and for the first time also cover the topic of dehydration, a very relevant, frequent and serious aspect of malnutrition in older persons.

Central recommendations of the ESPEN guideline for the management of malnutrition and corresponding evidence are summarised in Table 1 and described in the following Section 2.2.1, Section 2.2.2, Section 2.2.3, Section 2.2.4, Section 2.2.5, Section 2.2.6 and Section 2.2.7.

#### 2.2.1. Basic Recommendations

It is common sense that, as a first step in the management of malnutrition, those affected and also those at risk need to be identified. Thus, routine-screening for (risk of) malnutrition is recommended in all older people in institutions and in the community (at admission/initial contact and at regular intervals) independent of their diagnosis and the presence of overweight or obesity [21]. The use of a validated tool is considered to be good clinical practice, confirmed by recent study results indicating an association of the use of validated tools with lower prevalence of malnutrition and better nutritional care in hospitals [24]. The most common screening tool developed and validated for older persons is the short-form of the Mini Nutritional Assessment (MNA), which can be applied in all geriatric settings [2,21,25]. There are, however, many other tools available. Among 48 tools used to screen for risk of malnutrition in older adults, and recently rated with respect to validation, parameters and practicability, the highest scoring tools were: i) DETERMINE your health checklist for the community setting; ii) the Nutritional Form for the Elderly (NUFFE) for the rehabilitation setting; iii) the Short Nutritional Assessment Questionnaire-Residential Care (SNAQ^RC^) for residential care and iv) both the Malnutrition Screening Tool (MST) and the Mini Nutritional Assessment Short Form Version 1 (MNA-SF-V1) for the hospital setting [26].

In people with a positive screening result, a comprehensive nutritional assessment should follow as the basis for targeted interventions [21]. The assessment should focus on the identification of potential underlying causes of malnutrition as well as of individual preferences, resources and expectations, evaluation of the severity of the nutritional deficit, and a critical review of existing dietary prescriptions. To check whether intervention goals have been reached, a close monitoring is necessary in clinical practice [21].

Regarding interventions, due to the huge heterogeneity of older people and the multitude of potential causes of malnutrition [14,15], individualised and comprehensive approaches are recommended to optimally tackle malnutrition [21]. Beneficial effects on several outcomes are documented in quite a few randomised controlled trials, interestingly all reporting benefits with respect to quality of life [21].

It is recognised that identifying and eliminating potential causes as far as possible is fundamental, although scientific evidence for this recommendation is unfortunately lacking. In older patients, adequate medical treatment is certainly of central importance, preferably avoiding medication with potentially harmful side effects on appetite, taste and smell perception, salivation or cognition.

Comprehensive approaches and treatment focused on potential causes from all areas of life require the involvement of different professional disciplines namely dietitians, nurses, nurse-aids, kitchen staff, medical doctors including dentists, and all types of therapists (e.g., speech-/swallowing, occupational, physio- and psycho-). This team effort is regarded an important factor for successful nutrition interventions. Positive effects on body weight, functional and clinical outcome have been shown in several trials, although results are partly inconsistent [21].

#### 2.2.2. Supportive Interventions

Several recommendations in the ESPEN guideline address supportive interventions, which were highlighted more than 15 years ago in the resolution of the Council of Europe on food and nutritional care in hospitals [27]. Beneficial effects of assistance with eating, as far as required, and of a pleasant eating environment in institutions on dietary intake of older persons with malnutrition or at risk of malnutrition are well documented in several systematic reviews. A home-like dining environment has been shown also to contribute to quality of life. Based on expert consensus, it is also recommended to encourage older persons to share their mealtimes with others, since eating in company is known to stimulate dietary intake and may also be an important aspect with respect to quality of life [21].

In addition, the ability to access the meal (in case of mobility limitations) and the food (e.g., in packages that are difficult to open) may be relevant [28]. Of course, food should be easily accessible—also between meals—and support with shopping and preparing meals, reaching the dining room and opening packages should be provided as needed. In individual cases, it may be sensible to provide specially adapted cutlery or cups with special shapes.

The need for education regarding nutrition among all staff groups and the need for knowledge among patients about the importance of a good nutritional status are well recognised [27]. Several systematic reviews document that older persons with malnutrition or who are at risk of malnutrition, as well as their care givers, should be offered nutritional information and education as part of a comprehensive intervention concept in order to improve awareness of and basic knowledge about nutritional problems, and thus promote adequate dietary intake, albeit based on partly conflicting and fragmentary evidence [21].

#### 2.2.3. Nutritional Counselling

Nutritional counselling goes beyond information and education with the aim to develop a sound understanding of nutritional topics and to support sustainable health-promoting eating habits, and is regarded as the first line of nutrition therapy. Current guidelines recommend that older people with or at risk of malnutrition and/or their caregivers should be offered individualised nutritional counselling by a qualified dietician in several sessions to develop their understanding of the importance of nutrition and support healthy eating habits [21]. Individual sessions may be combined with group sessions, telephone contacts and written advice.

Counselling by a dietician or nutritionist is recommended, but in practice this may be impractical for all and reserved for those patients at highest risk. Many older people at risk of malnutrition live at home and will have initial contact with their primary healthcare team who could review their nutritional status and deliver appropriate advice, supported by local care pathways that include dietetic referral where appropriate [29]. However, it is unclear how to train these healthcare teams to best deliver the nutritional counselling. Group educational sessions for older people can be cost-effective but difficult to access for those with limited mobility. Older people may have difficulty accessing advice delivered in electronic formats but telephone consultations can effectively offer nutritional support for older people [30] and for family carers of those with dementia at risk of malnutrition [31].

#### 2.2.4. Food Modification

Food modifications include adjustments of macro- and/or micronutrient content, or the avoidance of specific allergens as well as modifications of food texture or of flavor, taste and/or visual appearance (organoleptic enhancement). Nutrients or additional ingredients can be added to regular foods in order to increase energy and/or nutrient density (fortified or enriched food) or to yield specific beneficial health effects (functional food) [32].

It has been shown in several studies and summarised in two systematic reviews that food fortification—i.e., by means of natural foods (e.g., oil, cream, butter, eggs) and/or specific nutrient preparations (e.g., maltodextrin, protein powder)—can enable increased intake while eating similar amounts of food [21]. Snacks between meals and/or finger foods can also help to increase intake, in particular for people who have difficulties using cutlery or remaining at the table for the meal; this is, however, not well studied [21].

Texture-modified foods are available in various qualities (e.g., liquidised/thin puree, thick puree/ soft and smooth, finely minced) [32] and intend to compensate for chewing and swallowing problems, which are widespread in older people and related to poor food intake [33]. Since evidence regarding the effects of texture-modified food is scarce [21], it was concluded that it is ‘good clinical practice’ to offer texture-modified, enriched foods to older persons with malnutrition or at risk of malnutrition and signs of oropharyngeal dysphagia and/or chewing problems as a compensatory strategy to support adequate dietary intake [21,34]. Based on positive effects of enrichment of regular texture diets it was assumed that enrichment could have similar effects in texture-modified diets for patients with chewing and/or swallowing problems [21]. Since insufficient dietary and fluid intake is described in older people receiving texture-modified diets, it seems reasonable to monitor nutritional intake closely [21].

Due to little expense and no risk of harm, these recommendations were made despite presently very limited scientific evidence [21].

In addition, it seems logical that increasing the variety of foods offered and considering individual food preferences could help to ensure adequate intake of older persons—this is however not studied up to now and not addressed in current guidelines.

#### 2.2.5. Oral Nutritional Supplements (ONS)

Several recommendations of the ESPEN guideline on clinical nutrition and hydration in geriatrics address whether older people with malnutrition or at risk of malnutrition should be offered ONS. Such supplements provide both macro- and micronutrients which are delivered as ready to drink liquids, or as semi-solids or powders that can be prepared as drinks or added to drinks or foods. According to a vast body of high-level evidence and strong consensus among experts, these supplements should provide at least 400 kcal and a minimum of 30 g of protein per day. They should be given to all older people with (risk of) malnutrition when nutritional goals cannot be met through dietary counselling for enhancing (fortified) food consumption, to improve dietary intake, body weight and to lower the risk of complications and readmission and to lower the risk of functional decline after discharge. Once offered, ONS should be given for at least one month with concurrent monthly assessment of presumed benefits and compliance evaluation, thereby tailoring ONS-type, flavour, texture and timing of supply to the older person’s characteristics [21].

#### 2.2.6. Enteral and Parenteral Nutrition

Enteral nutrition (EN)—mostly via nasogastric tubes or percutaneous endoscopic gastrostomy (PEG)—and parenteral nutrition (PN) via central or peripheral veins are important options also for old and very old patients. These invasive measures should however be reserved for those who are unable to meet their nutritional requirements by the oral or enteral route, respectively, but have a reasonable prospect of general recovery or at least stabilisation of health and well-being. Twelve recommendations in the ESPEN guideline refer to this topic, all based on available descriptive studies and expert consensus, since randomised trials would be unethical in this field. Application of these techniques always requires careful weighing of expected individual benefits and risks [21].

#### 2.2.7. Relevance of the Refeeding Syndrome (RFS)

Based on recent research activities, management of malnutrition in older persons cannot be described without pointing out the RFS, a serious metabolic complication after reinitiating nutrition in malnourished patients [35,36]. If patients with RFS are not adequately treated, adverse effects may range from muscle weakness and peripheral oedema to multi-organ dysfunction and death [36].

The risk of developing RFS is suggested to be high especially among malnourished older patients, and is not restricted to enteral or parenteral nutrition. However, due to nonspecific initial symptoms [36] but also due to a lack of knowledge among many physicians [37], the RFS is frequently not diagnosed and consequently not treated in these patients [38]. A recent cross-sectional multicentre-study showed that nearly three-quarters of 342 geriatric hospitalised patients who were at risk of malnutrition demonstrated significant risk of RFS [39]. Like malnutrition, RFS remains a widely unrecognised and undertreated condition in clinical practice.

The key to improved patient care in this context is to raise awareness of RFS among physicians involved in nutritional care in order to identify at-risk patients and to recognise the occurrence of the RFS. In the ESPEN guideline, it is recommended to pay special attention during the first three days of EN and PN therapy in malnourished individuals to serum levels of phosphate, magnesium potassium and thiamine, which decline in RFS and should be supplemented where appropriate [21]. Accordingly, a recent review [38] also recommends close monitoring of vital parameters, fluid, serum electrolytes and thiamine in older patients at risk of RFS, whereas nutrition repletion should be started slowly and increased cautiously to reach nutritional goals after four to seven days. Using this strategy in a randomised clinical trial, mortality risk was reduced among critically ill patients [40].

### 2.3. New Evidence after the ESPEN Guidelines

After the literature review for the ESPEN guideline was completed, a number of new systematic reviews and clinical trials addressing different nutritional interventions in different setting have been published.

A systematic review and meta-analysis considered studies that only included older persons with malnutrition and found no beneficial effects of ONS in changing body weight, body mass index, MNA score, muscle strength, activities of daily living, Timed Up&Go test, quality of life and mortality. Results of other interventions (dietary counselling and ONS, ONS combined with exercise, new ONS nutrition delivery systems) were inconsistent [41]. Another systematic review that explored the role of exercise added to oral nutritional support reported improvements in muscle strength but not in any other outcome, however mostly based on low or very low quality evidence [42]. An interesting analysis showed that nutritional support performed by a multidisciplinary team—as recommended in the ESPEN guideline—might fare better than simple interventions in reducing mortality risk and improving quality of life [43]. A systematic review looking at studies performed in nursing homes found an effect on handgrip strength only and not in other functional parameters [44]. A further recent review focused on various treatments for anorexia of ageing, also including pharmacologic approaches and flavour enhancement, suggests that some interventions may have an impact on energy intake and body weight, but calls for methodological improvements in the field [45]. Based on a systematic review and meta-analysis, the use of telehealth interventions seems to be a new, promising strategy also for malnutrition in older people. Improved protein intake and quality of life are reported, but further research is demanded [30].

Thus, the number of systematic reviews is rapidly increasing with only few remarkable original studies. Among these, a pooled analysis of individual data from nine RCTs in older adults at risk of malnutrition merits attention. Positive intervention effects on energy intake and body weight were found, whereby the combination of nutritional counselling and ONS showed the strongest effects [46]. Pooled data from studies targeting muscle strength and mortality, however, revealed no intervention effect [47]. In contrast, the recently published large multicentre EFFORT trial demonstrated beneficial effects on important clinical outcomes by routine malnutrition screening connected with individualised nutrition support in medical inpatients managed by a dietician during hospital stay [48].

## 3. Knowledge Gaps

### 3.1. Lacking Evidence in Many Fields

Despite increasing numbers of systematic reviews on the topic, more than half of the recommendations in the ESPEN guideline are “good practice points”, meaning that they are based on the clinical experience of the guideline development group because of a lack of studies [49]. The level of evidence of only 15 of the 82 recommendations justifies a grade A recommendation, and only three recommendations are directed towards patient-centered outcomes [21].

Regarding outcome parameters, most studies testing the effects of supportive interventions or food modification focus on dietary intake but do not include functional or clinical outcomes.

Most randomised clinical trials performed have examined the effects of ONS in (malnourished) older adults. Given the large number of studies and the generally quite good compliance (78%) [50], this study and intervention type currently provides the strongest evidence in the field. Besides positive effects on intake, positive effects on nutritional status are also reported, and a couple of studies also look at functional and clinical outcomes. Unfortunately, findings in this latter respect are inconsistent and often negative [21], and benefits were generally questioned just recently [41].

A general lack of scientific evidence on the effects of nutritional interventions on functional or clinical outcomes in malnourished older adults is also concluded in multiple systematic reviews [21,41,51,52,53,54,55].

### 3.2. Limitations of Existing Studies

**Figure 1:**
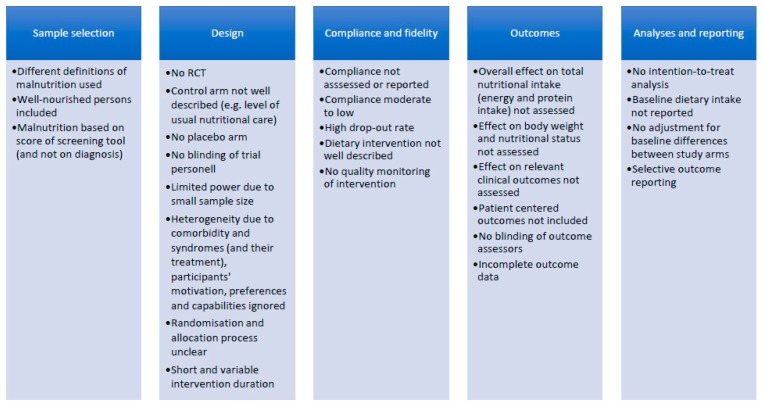
Limitations of previous studies investigating the effect of nutritional interventions in malnourished older people.

The conflicting evidence regarding effects of nutritional interventions on functional and clinical outcomes of malnourished older adults may be explained by various limitations of previous studies. The limitations frequently addressed in the literature are summarised in Figure 1, categorised according to sample selection, design of the study, adherence to the intervention, outcome assessment, and data analyses and reporting. Some of these limitations result in a high risk of bias [56,57], thereby reducing the internal validity of previous studies.

With regard to sample selection, some previous studies have not only included malnourished persons but older adults without malnutrition as well, which likely dilutes the potential effect of treatment on outcomes [55]. Different definitions of malnutrition were used to select participants, reducing comparability between studies. Among frequently occurring limitations of study design are a small sample size and/or lack of a proper power calculation based on a primary outcome variable, thus limiting the power to detect an effect. Baseline nutritional intake is usually not reported. In studies using ´usual care´ in the control arm, the level of nutritional care is mostly not or only poorly described, and thus the contrast between control and intervention arm remains unclear. Hence, possible differences are not attributable to the specific intervention but rather to the difference in the overall quality of nutritional care. Further, participants and study personnel are often not blinded leading to performance bias. Even in situations where a placebo intervention appears possible (for example using a low caloric, similar looking supplement when ONS is being tested) placebo is rarely used. In most studies, a single intervention is used for all selected participants, without incorporating knowledge of participants’ health and functional status, other treatments, and participants’ motivation and preferences, which may reduce its effect. Details regarding compliance and intervention fidelity are sometimes not reported, while low compliance and poor fidelity will likely reduce potential effects. With regard to study outcomes, some studies lack the inclusion of clinically relevant or patient-centred parameters such as quality of life. Detection bias is often present due to non-blinding of the outcome assessors [56,57]. Moreover, studies often describe the provided amounts of energy and protein but do not report the net effect on the overall daily intake (which may have increased less due to energy compensation at other meal times) [46]. Finally, when analysing the results, several previous studies did not perform intention-to-treat analyses, but used complete case analysis only, possibly inducing further bias.

Overall, due to one or more of these limitations in most of the studies, the available evidence is mostly of only low or moderate quality. Moreover, due to large heterogeneity of inclusion criteria and study populations, intervention types and outcome assessments, the pooling of individual patient data and performing meta-analyses is currently seriously hampered [47,57]. Applying a fixed definition for malnutrition in future studies could importantly increase comparability between studies. The field could also benefit from establishing a minimum dataset (MDS) for clinically relevant outcome variables, per setting when deemed necessary, and defining the preferred method for assessing these outcome variables. This MDS would stimulate the incorporation of standardised outcome variables in future trials, and would enable the pooling of data and the performance of meta-analyses in order to obtain the highest level of evidence regarding the effect of nutritional interventions on relevant clinical outcomes.

### 3.3. Open Questions and Research Needs

In view of the recent global malnutrition definitions, which also consider muscle mass and inflammation as part of malnutrition [8], an update of prevalence data using this definition is needed.

Validity of available screening tools remains unclear due to multiple methodological problems [58]. Setting-specific tools have been suggested based on a newly developed scoring system which was applied to 48 existing tools, but more work is still needed to derive sound recommendations regarding the optimal tool [26].

Based on limited and partly conflicting evidence and limitations of existing studies outlined above, it remains unclear which interventions are most effective in which patient groups, and if specific situations (e.g., acute malnutrition), specific diseases (e.g., dementia) or specific etiologies of malnutrition require specific approaches. Interestingly and for example, an animal study with an infection-triggered model of acute malnutrition has recently demonstrated differential effects of parenteral glucose supplementation in addition to free oral intake on survival: Whereas in mice with an experimental viral infection a significant survival benefit was observed, mortality was significantly higher in mice with an experimental bacterial infection [59]. Thus, it may be hypothesised that especially in acute malnutrition nutrition support is not always beneficial or might need a specific approach.

With regard to a causative treatment, the complex etiology of malnutrition needs further research. The etiologic relevance of the many different potential causes and therefore the priority of distinct diagnostic measures remains unclear, and a treatment, based on individually identified causative factors is probably only rarely performed. More knowledge about the most relevant causes and their common pathophysiology would increase the potential for causation-oriented treatment and could in addition contribute to enhance malnutrition awareness and preventive approaches in patients with the respective problems.

Regarding general intervention characteristics, individualised, multimodal and multi-disciplinary strategies seem to be promising and should be pursued further.

In terms of specific interventions, food fortification, additional snacks and finger food are promising options but need high-quality reassessment through further studies that provide reliable evidence. Possible beneficial effects of all different types of food modifications including also organoleptic enhancement or texture modifications need to be tested in explorative trials as well as in subsequent sufficiently powered high-quality trials.

Further research is also needed to determine which methods of delivering dietary counselling are appropriate and cost-effective for different participants and care settings. It is also unclear whether and if when the counselling should be repeated and followed up. Furthermore, the role of social care staff, such as domiciliary carers, in delivering nutritional advice and support should also be explored.

Moreover, RFS must be highlighted as an area that needs further high-quality research to develop the best preventive strategies as effective treatments for malnutrition.

Relevant outcomes for malnutrition intervention studies were recently agreed in a Delphi study among geriatric and nutrition experts [57], however more research is also needed in this regard. Clearly, patient-centred outcomes such as independence in activities of daily living and quality of life need much more attention than in the past and should be assessed using reliable and meaningful tools. Also, health and social care resource use and cost-effectiveness are important outcomes which need to be further addressed. Here, a comprehensive, cross-sectoral perspective is needed as treatment costs in one setting may only be offset by a larger cost saving in another setting.

As studies were mainly conducted in the hospital setting, further research in other health-care settings is needed.

Furthermore, the topic of dehydration urgently needs further research efforts. As one of the most frequent diagnoses of older patients, the issue of dehydration has been addressed in the current ESPEN guidelines [21], which however also uncovered many open questions and lacking evidence. For example, there is no uniform definition of dehydration, the diagnostic criteria remain vague and there is no accepted screening tool. Effective strategies to better prevent dehydration must be developed.

Last but not least, effective ways to implement current knowledge in clinical practice need to be explored.

These and other topics considered important by the authors are listed in Table 2.

## 4. Conclusions

In conclusion, we are faced to a wide range of unresolved issues regarding the management of malnutrition in older persons which need to be addressed. Many of these questions cannot easily be answered, and it is an important next step to develop innovative strategies and well-conceived concepts for this purpose. Altogether, high-quality research is urgently required to develop effective strategies for the prevention and treatment of malnutrition in the increasing number of old and very old patients at risk.

## Figures and Tables

**Table 1:** Intervention strategies for the management of malnutrition (based on [21]).

**Basic recommendations** Routine screening for malnutrition with validated tool (GPP) followed by assessment, individualised intervention, monitoring and adjustment of interventions (GPP) Individualised and comprehensive nutritional care (A) Nutritional interventions as part of a multimodal and multidisciplinary team intervention (B) Identification and elimination of potential causes of malnutrition (GPP) Avoidance of dietary restrictions (GPP)
**Supportive interventions** Pleasant eating environment in institutions (A) Mealtime assistance in case of eating dependency (A in institutions, GPP in home-care) Sharing mealtimes with others (GPP) Energy-dense meals on wheels with additional meals (B) Nutritional information and education (B) Easy access to food *
**Nutritional counselling** for older persons/care givers—individualised (B) by a qualified person in several sessions (GPP)
**Food modification** food fortification (B) additional snacks/meals*, finger food (GPP) texture-modified, enriched foods (GPP) organoleptic enhancement (flavor/taste/visual appearance) * increasing variety of diet * considering individual preferences *
**Oral nutritional supplements (ONS)** (3 A, 3 GPP)
**Enteral/parenteral nutrition** (12 GPP)

Grades of recommendation: A = based on strong evidence (at least one high-quality RCT), B = based on medium evidence (high quality case-control or cohort studies); GPP = good practice point/expert consensus: Recommended best practice based on the clinical experience of the guideline development group.* topic not addressed in the ESPEN Guideline 2019 [21]).

**Table 2:** Open questions regarding management of malnutrition in older people.

**Prevalence, screening and diagnosis** How are prevalence data affected by the new global definition of malnutrition (GLIM criteria)? Are the new GLIM criteria appropriate for older persons? Are different screening tools needed in different care settings? Which screening tools should be used in which setting? Are there biomarkers that could enhance screening and diagnosis of malnutrition?
**Determinants and multifactorial etiology of malnutrition** What are the most relevant causes of malnutrition in older patients? What are the etiologic mechanisms? What is the role of medication in malnutrition and reduced appetite? What are the essential aspects of assessment of the causes of malnutrition in an individual?
**Effectiveness and safety of interventions** Which interventions are most effective in which patient groups? Should specific interventions have priority? What are the best outcomes to assess the effect of interventions? Which interventions are most cost-effective? What is the optimal duration of interventions in each health-care setting? Do specific situations (e.g., acute malnutrition), specific diseases (e.g., dementia) or specific etiologies of malnutrition require specific intervention approaches? Are there situations, e.g., acute disease, where increasing energy intake could be harmful? Which strategies are most effective to prevent RFS but effectively treat malnutrition at the same time? Which types of food modifications are beneficial? Which methods of delivering nutritional counselling are appropriate and cost-effective for different participants and care settings? (When) should nutritional counselling be repeated and followed up? What is the role of social care staff in delivering nutritional advice and support? At what degree of malnutrition do patients benefit from interventions? At what degree of malnutrition do patients benefit from (par)enteral nutrition? Which interventions are effective with respect to patient-centred outcomes? Do malnourished obese patients need a specific approach?
**Natural recovery** Are there patients who do not need treatment because of early natural recovery? Can early natural recovery be predicted?
**Role of protein and other specific nutrients** How much protein is required in specific situations (e.g., diseases, nutritional states, functional states)? How should protein intake be distributed over the day? Is there an optimal time of protein intake in relation to physical training? What is the relevance of different protein sources for meaningful outcomes? Which micronutrients are frequently deficient in malnourished older persons? Is supplementation of any micronutrient beneficial in malnourished older persons?
**Dehydration** What are reliable diagnostic criteria for dehydration? How could an effective screening for dehydration be performed? How is the overlap of dehydration with malnutrition? What are effective preventive approaches against dehydration?
**Knowledge transfer into clinical practice** How can knowledge about malnutrition and respective guidelines be effectively implemented in clinical practice? What are the effects of nutritional training for health care professionals?

GLIM = Global Leadership Initiative on Malnutrition.
